# Obstetric Ultrasonography to Detect Fetal Abnormalities in a Mouse Model for Zika Virus Infection

**DOI:** 10.3390/v12010072

**Published:** 2020-01-07

**Authors:** Dominik Forster, Jan Hendrik Schwarz, Katrin Brosinski, Ulrich Kalinke, Gerd Sutter, Asisa Volz

**Affiliations:** 1 Institute for Infectious Diseases and Zoonoses, Ludwig-Maximilians-Universität Munich, 80539 Munich, Germany; dominik.forster@micro.vetmed.uni-muenchen.de (D.F.); jan.schwarz@micro.vetmed.uni-muenchen.de (J.H.S.); katrin.printz@micro.vetmed.uni-muenchen.de (K.B.); gerd.sutter@lmu.de (G.S.); 2 Institute for Experimental Infection Research, TWINCORE, Centre for Experimental and Clinical Infection Research, a joint venture between the Helmholtz Centre for Infection Research Braunschweig and the Hannover Medical School, 30625 Hannover, Germany; 3 German Center for Infection Research (DZIF), partner site Munich, 80539 Munich, Germany

**Keywords:** Zika virus, uteroplacental infection, viral pathogenesis, pregnancy, ultrasound

## Abstract

In 2015 Zika virus (ZIKV) emerged for the first time in South America. The following ZIKV epidemic resulted in the appearance of a clinical phenotype with microcephaly and other severe malformations in newborns. So far, mechanisms of ZIKV induced damage to the fetus are not completely understood. Previous data suggest that ZIKV may bypass the placenta to reach the fetus. Thus, animal models for ZIKV infection are important to facilitate studies about ZIKV infection during pregnancy. Here, we used ultrasound based imaging (USI) to characterize ZIKV induced pathogenesis in the pregnant Type I interferon receptor-deficient (IFNAR-/-) mouse model. Based on USI we suggest the placenta to be a primary target organ of ZIKV infection enabling ZIKV spreading to the fetus. Moreover, in addition to direct infection of the fetus, the placental ZIKV infection may cause an indirect damage to the fetus through reduced uteroplacental perfusion leading to intrauterine growth retardation (IUGR) and fetal complications as early as embryonic day (ED) 12.5. Our data confirmed the capability of USI to characterize ZIKV induced modifications in mouse fetuses. Data from further studies using USI to monitor ZIKV infections will contribute to a better understanding of ZIKV infection in pregnant IFNAR-/- mice.

## 1. Introduction

ZIKV has been first identified in 1947 in a sentinel monkey in ZIKV forest in Africa. First confirmation of ZIKV in humans has been obtained in 1952, when ZIKV was firstly isolated from a human suffering from the so called Zika Fever [1]. Since then, sporadic ZIKV outbreaks have occurred in Africa and Asia [2]. Another clinical outcome of ZIKV infection is the induction of the Guillain-Barré syndrome manifesting in various neurological disorders through damaging the peripheral nervous system [3,4,5,6]. As a classical arbovirus, ZIKV is transmitted by mosquitos [7,8,9]. In addition, recent data confirmed that ZIKV can be also spread through the sexual and vertical route [10,11,12,13] In 2015 the virus suddenly emerged on the American continent causing an epidemic of congenital human abnormalities. During the epidemic, ZIKV infection during pregnancy resulted in fetal abnormalities such as microcephaly, neurological malfunction, and spontaneous abortion [11,14,15]. Of note, a significantly increased occurrence of the congenital ZIKV syndrome (CZS) has been observed in South America in Brazil, Columbia and other countries, with diagnosis occurring in different geographical areas without epidemiological connectivity. Interestingly, in a cohort study performed early during the Brazilian ZIKV epidemic revealed that 42% of the fetuses developed CZS independent of the trimester of infection in a cohort of 182 ZIKV-infected and symptomatic pregnant women [14]. In human fetal studies brain imaging findings have been undertaken using magnetic resonance imaging (MRI) and fetal ultrasound [16,17,18]. The major clinical findings during the pregnancy included ventriculomegaly in the cerebrum, hypoplasia of the cerebellum, calcifications and myelination defects resulting in different malformations [19,20]. Moreover, after birth CZS is characterized by significant damage of the sensorineurological outcomes including hearing loss and significant ocular defects [21,22,23,24]. However, we still know relatively little about the specific pathological mechanism underlying CZS [25]. Preliminary insights into the molecular mechanism of ZIKV infection within fetuses came from studies using in vitro cultures of different neuronal progenitor cells and brain cells [26]. Here, ZIKV infection of progenitor cells of the cerebral cortex increased inflammation and cell death resulting in reduced cell proliferation. Moreover, ZIKV infection of astrocytes induced the modulation of the specific immune responses that suppresses the cell surface protein Axl [27,28]. Another mechanism of ZIKV induced feto-pathogenesis as identified in an infection model of brain slices in vitro generated from embryonic mice of various ages showed that ZIKV replication in the neocortical proliferative zone and in the developing midbrain induced apoptosis, which may limit viral dissemination in the brain and may constrain virus-associated injury [29]. In addition, the placenta is an important target organ of mother to fetus derived ZIKV infection. Here, in vitro evaluation of placental cell organoids revealed that human trophoblast isolated early during pregnancy propagated ZIKV to very high titers [30,31]. In contrast, ZIKV infection of human trophoblast derived from later pregnancy stages demonstrated less efficient ZIKV amplification compared to the early pregnancy [32]. This result may further support the hypothesis of reduced susceptibility of fetal ZIKV infection in later stages of pregnancy [33]. Currently, it is not understood how likely ZIKV infection during pregnancy results in infection and damage to the fetus. So far, the first trimester of human pregnancy is considered as the time period in which the fetuses are most vulnerable to ZIKV infection and the development CZS [34,35,36]. After confirmed or possible ZIKV infections in pregnant women, the Center for Disease Control and Prevention (CDC) recommends repeated fetal ultrasound to monitor for possible fetal malformation especially in the head regions. For humans, fetal ultrasound is well established and easy to interpret due to a wide range of technical features and support. However, with the progression of the American ZIKV epidemic, the incidence of ZIKV infection in pregnant women is also declining, before a detailed understanding of CZS has been accomplished. Therefore, animal models of ZIKV infection are important and essential to better understand the mechanisms and causes of CZS. Non-human primates have been established as a model for maternal ZIKV infection during pregnancy. In that context, Hirsch and coworkers already successfully used non-invasive ultrasound (US) throughout pregnancy to evaluate fetal growth, uteroplacental blood flow and amniotic fluid index [37]. The mice lacking the interferon alpha/beta receptor (IFNAR-/-) provide a promising preclinical model of congenital ZIKV infection. Here, challenge infection on embryonic day 7.5 (ED 7.5) results in fetal abnormalities resembling the intrauterine growth restriction and spontaneous abortion observed in ZIKV-infected pregnant women. For this, the development of fetal ultrasound diagnostic tools might be advantageous to better understand the pathogenesis of CZS in an early stage of mouse pregnancy. In addition, this approach would be helpful to test new therapies and vaccines that could prevent or mitigate the intrauterine ZIKV infection. Here, we characterized the outcome of ZIKV infection in pregnant IFNAR-/- mice using ultrasound imaging (USI). We confirmed the occurrence of ZIKV induced malformations and damage to fetuses on ED 13.5. Interestingly, modifications in the placenta, a major target organ of ZIKV infection, were not clearly visible by USI before ED 14.5. As the first sign of ZIKV induced pathogenesis we detected evidence of disturbed blood circulation in the uteroplacental vessels as early as ED 12.5 supporting the hypothesis of hypoxia induced damage to fetuses. These results support the future use of USI to better characterize ZIKV pathogenesis in pregnant IFNAR-/- mice.

## 2. Materials and Methods

### 2.1. Study Design

Female 6-10-week-old IFNAR -/- mice were mated in a 1:1 scheme for 24 h. Successful mating was observed by plug check on the following day. Plug positive mice were then separated and examined via ultrasound starting on embryonic day (ED) 4.5 which is the first timepoint for reliable confirmation of pregnancy. Signs of illness, weight loss, and survival were monitored daily. In two independent experiments mice (*n* = 10) were infected on ED 7.5 with a total dose of 10^3^ plaque-forming-units (PFU) of Zika virus strain H/PF/2013 (EVAg, clinical isolate, French Polynesia 2013, GenBank Sequence Accession: KJ776791) diluted in 50µl physiological saline via footpad infection in the left hind leg. Control mice (*n* = 8) were mock-infected with saline (PBS).

On ED 16.5 mice were euthanized and fetuses (*n* = 7 0), placentas (*n* = 7 0) and selected maternal organs (brain, spinal cord, uterus, and ovaries) were harvested, weighed and documented. After fixation of samples with paraformaldehyde for histopathology, remaining organ material was stored at −80 °C for titration of infectious virus.

### 2.2. Mice

IFNAR-/- mice [38] backcrossed more than 20-fold on the C57BL/6 background were bred under specified-pathogen-free (SPF) conditions, housed in isolated cage units (IsoCage, Tecniplast, Hohenpeißenberg, Germany) and had access to food and water ad libitum. All experiments were approved by the Government of Upper Bavaria, Munich Germany and were performed in compliance with the German Animal Welfare Act.

### 2.3. Cells and Viruses

Vero (African green monkey kidney) cells (ATCC CCL-81) were used. Plaque-purified Zika virus (ZIKV) Isolate H/PF/2013 (EVAg, clinical isolate, French Polynesia 2013, GenBank Sequence Accession: KJ776791) was propagated on Vero cells. Viral titers were determined by plaque assay and titrated, with values reported in PFU as previously described [39].

### 2.4. Ultrasound Imaging

**Figure 1:**
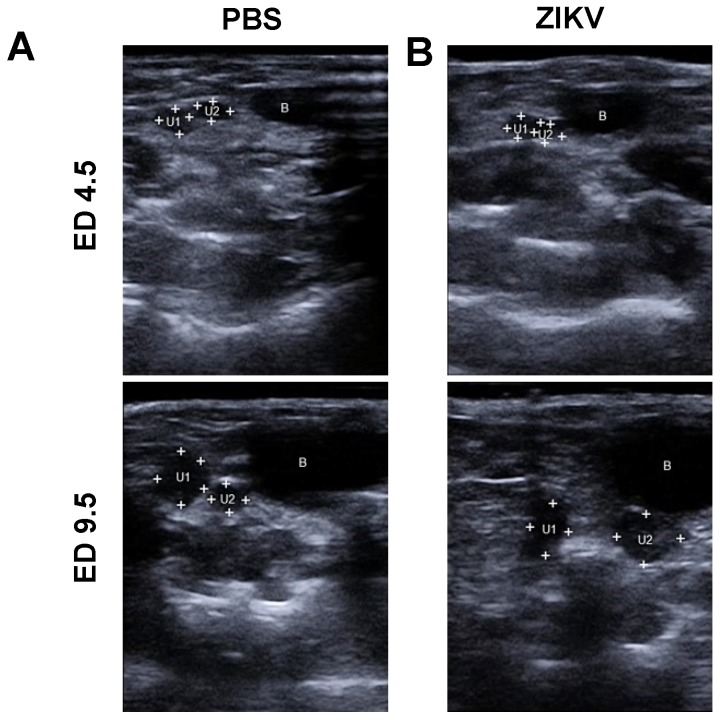

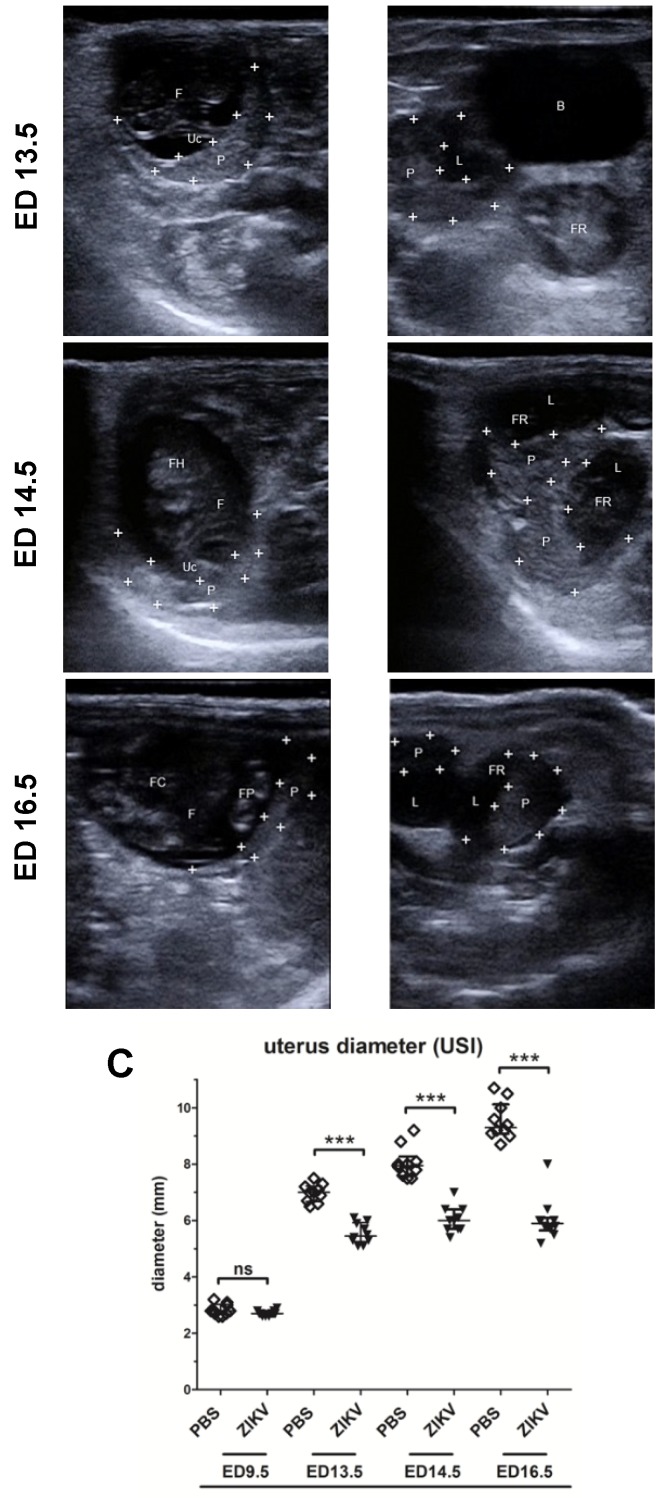

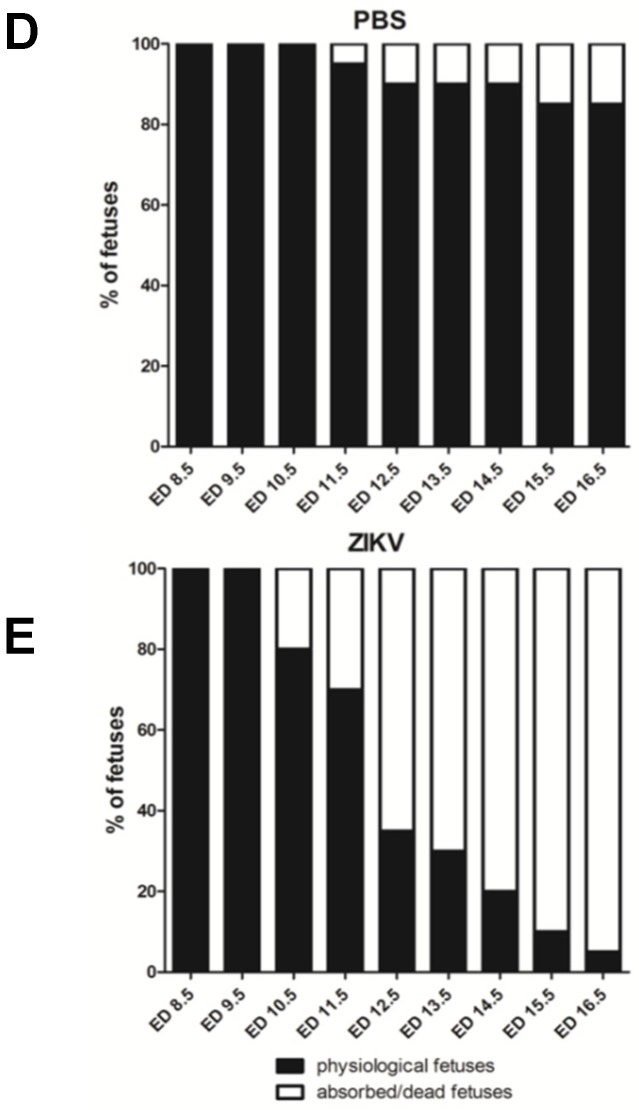
Embryonic day (ED) 4.5: (**A**) + (**B**) A two-dimensional echocardiography image of the uterus showing the implantation site (uterus horn 1 (“U1”) and uterus horn 2 (“U2”) framed by white crosses) next to the bladder (“B”, shown in black) on ED 4.5.; ED 9.5: (**A**) + (**B**) Echogenic foci of the uterus horns are prominent on ED 9.5.; ED 13.5: (**A**) the placenta and the developing fetuses are visible (“P”, framed by white crosses, “F”) and the umbilical cord (“Uc”), (**B**) the placenta is visible in the uterus lumen (“P”, framed by white crosses, “L”, shown in black) and indistinguishable fetal structures (“FR”); ED 14.5: (**A**) growing fetus (“F”), with distinct structures such as the fetal head (“FH”), the umbilical cord (“Uc”) and the placenta (“P”, framed with white crosses) on ED 14.5, (**B**) the uterus lumen is either filled with fetal residues including the placentas (“FR”, “P”, framed with white crosses) or empty on ED 14.5; ED 16.5: (**A**) highly developed fetus with distinguishable fetal thorax, the fetal heart and paws (“F”, “FC”, “FP”) and the placenta (“P”, framed with white crosses) on ED 16.5, (**B**) Uterus lumen (“L”, shown in black) including placentas with unclear fetal residuals on ED 16.5; (**C**) Analysis of uterus diameter using USI at different timepoints during pregnancy (*n* = 8- 10dams per group, graph represents 2–3 independent measurements per uterus). Differences between individual groups were analyzed by Mann–Whitney test. Error bars indicate the interquartile range (IQR) from the median. Asterisks represent statistically significant differences between two groups: ns = non-significant, *** *p* = 0.0002; (**D**) Tabular summary of the ultrasound based findings as identified in (**A**,**B**) in the mock-infected mice (*n* = 20 fetuses, 4 examined fetuses/uterus ampoules per mouse and timepoint); (**E**) Tabular summary of the ultrasound based findings as identified in (**A**,**B**) in the Zika virus (ZIKV)-infected mice (*n* = 20 fetuses, 4 examined fetuses/uterus ampoules per mouse and timepoint).

For ultrasonographic examination we used the ultrasound system MyLab^™^Delta (Esaote, Genoa, Italy) with a linear array 20 MHz transducer (SL3116, Esaote, Genoa, Italy). We chose an ultrasound system that is routinely used in veterinary medicine for multiple approaches. Unanesthetized pregnant mice were exposed to B-Mode, 20MHz, pulsed USW and a scanning rate of 35 frames per second for 2-3– min in different schedules dependent on total duration and pregnancy time points (Figure 1). Approximately 1 cm^3^ prewarmed Aquasonic ultrasound gel (Parker, Fairfield, NJ 07004 USA) was placed between the transducer face and the abdomen of the mouse. Mice were scanned from caudal to cranial. In doing so, we positioned the bladder and the uterus including the specific position of the fetuses. We used the standard ultrasound examination to assess the morphology and size of the fetuses and the placenta. In addition, standard Doppler ultrasound measurements evaluating uteroplacental hemodynamics were performed by using the MyLab^™^Delta as described before [40]. Color Doppler ultrasound complements the ultrasonic B-mode examination and enables the assessment of perfusion and circulation. Color Doppler ultrasound can be used to demonstrate the presence or the reduction of blood flow and its direction. Signals are reported and visualized via blue or red signals. Blood flow activity was measured by using the mean of the score of pulsation in brain vessel or umbilical cord diameter and the score of the percentage of living fetuses (see Table 1 below). Fetal heart rate was quantified by manually counting fetal heart contractions and so resulting Doppler USI signal.

### 2.5. Determination of ZIKV Loads in Mouse Organs

Organs (brain, spinal cord, ovaries, uterus, placenta, embryonic head) were removed under aseptic conditions from sacrificed mice (*n* = 8–10). The organs were immediately frozen and subsequently thawed, weighed, and homogenized using 0,1 g of organ material with 1 ml PBS in a microtube (Retsch Tissue Lyser MM300, Qiagen GmbH, Hilden, Germany). Tubes were centrifuged for 1 min at 1.500 rpm and 4 °C. Supernatants were taken and stored at −80 °C. Viral titers in organ supernatants were determined by plaque assay as described before [39] and indicated in PFU per 1 g organ material.

### 2.6. Histopathology and Immunohistochemistry

Placentas of sacrificed mice were routinely fixed in paraformaldehyde for 24 h and subsequently embedded in paraffin. Sections of 4 µm were stained with hematoxylin and eosin (HE) before being evaluated by light microscopy. Primary antibody for immunohistochemistry (IHC) was a rabbit anti-Flavi-E diluted (1:1000, absolute antibody, Ab00230-23.0, Oxford, UK). Placentas of PBS-inoculated mice served as negative controls. After deparaffinization, sections were blocked with hydrogen peroxide followed by diluted normal goat serum (30 min). Primary antibody incubation was done for 60 min at room temperature. Biotinylated secondary antibody was a goat-anti-rabbit antibody (1:200, Vector, BA-1000, Burlingame, CA, USA). Peroxidase-complexed avidin-biotin (ABC-HRP, Vector, PK-6100) and diaminobenzidine (DAB) were used for visualization and hemalaun as counterstain. Control experiments were performed by using chimeric rabbit IgG (abcam, AB172730, Cambridge, UK) as negative control and primary antibody on a mixed cell culture of ZIKV-infected and non-infected Vero cells. Von Kossa staining was used to detect and to quantify mineralization in placental samples. Images were taken by a Keyence microscope (Keyence BZ-X700, Keyence, Neu-Isenburg, Germany).

### 2.7. Statistical Analysis

All data were analyzed with GraphPad Prism version 5.0 (GraphPad Software Inc, San Diego CA, USA) and MedCalc software (MedCalc Software, Ostend, Belgium). Data were expressed as mean ± standard error of the mean (SEM) and as medians and the interquartile range (IQR) of the median. Statistical analysis to compare two groups was performed using the Mann–Whitney test, the unpaired, two-tailed t-test after calculation of the area under the curve and the Shapiro–Wilk test for normal distribution. The threshold for statistical significance was *p* < 0.05.

## 3. Results

### 3.1. Outcome of ZIKV Infection in Pregnant IFNAR-/- Mice as Analyzed by Sonography

To confirm pregnancy after positive plug-check, mice were examined on estimated ED 4.5 by ultrasound imaging (USI). Here sectional views (Figure 1, A+B, ED 4.5) showed substantial proliferation of both uterus horns (“U”) as seen in increased diameter relative to the bladder (“B”, shown in black). This increase is accompanied by proliferation of the mucosa as seen by higher echogenicity (image brightness in USI) on the sectional plane of the uterus. These observations confirmed the successful implantation process and mice that met these anatomical criteria as visualized by USI were considered positive pregnant and were included in this study.

These mice were analyzed by USI every day and representative images are shown in Figure 1 for EDs 4.5, 9.5, 13.5, 14.5, and 16.5. On ED 7.5 pregnant mice were either infected with ZIKV or with PBS as mock control. Two days after the challenge infection (ED 9.5) we observed continued growth of ampoules in both uterus horns, as seen by an average diameter of 2.8 mm for the PBS mock-infected mice (Figure 1 A, ED 9.5, “U1/2”, “B”, the bladder in black, Figure 1C) and an average in diameter of 2.6 mm in the ZIKV-infected mice (Figure 1B, ED 9.5, “U”, transverse sections in relative position to the bladder “B”, in black, Figure 1C). Of note, compared to the earlier embryonic stages as analyzed before, we succeeded in the imaging of two different sections through the uterus horn ampoules indicating an ongoing growth activity of physiological pregnancy for both experimental groups. These results indicate no obvious differences between PBS mock-infected mice and ZIKV-infected mice. On ED 13.5 USI identified a significant increase in the size of the uterus with a diameter of 7 mm in the mock-infected control group (Figure 1A, ED 13.5, Figure 1C). Of note bladder was not included in the picture using comparable insonation-angle (kept < 20°) supporting this observation. In addition, we were able to visualize highly echogenic structures (in USI brighter shades of grey) within the less echogenic (in USI darker shades of grey) liquid filled lumens of the uterus indicating a developing fetus (“F” in Figure 1A, ED 13.5).

A slightly brighter echogenic structure associated with the fetus suggested the starting formation of the first vessel structures. Under the tentative fetal structure another highly echogenic structure was visible that was identified as placental tissue (“P”, framed by white crosses). In ZIKV-infected animals we observed different uterus sections with a mean diameter of 5.5 mm indicating reduced fetal and placental development compared to the mock-infected control mice on ED 13.5 (Figure 1B, ED 13.5, Figure 1C). In addition, hypoechogenic structures within the uterus lumen demonstrated the presence of free liquids. Free liquids, identified as highly echogenic structures were also clearly detectable within the uterus lumen representing embryonic residues. Of note, bladder (“B”, black) still appeared in the USI while using the specific USI insonation-angle as established before. Another prevalent echogenic crescent-formed structure was visible on ED 13.5 in the ZIKV-infected mice showing the developing placental tissues (framed by white crosses).

On ED 14.5 USI in the PBS mock-infected group revealed the development of the ampoules with an average of 8 mm in diameter, corroborating the physiologic course of pregnancy (Figure 1C). This observation was further confirmed by transverse sections through the uterus horn lumens. Here, the specific echogenic structures strengthened the detection of a growing fetus (“F” in Figure 1A, ED 14.5), as identified by prominent anatomical structures, the fetal head (“FH”) and fetal limbs. Moreover, on ED 14.5 the specific vessel structure, first seen on ED 13.5, showed further consolidation and was identified as the umbilical vein (“Uc”), connecting the fetus to the mother. In line with this observation, we could also detect the consolidation of the placental tissue (“P”, framed by white crosses).

In contrast, in the ZIKV-infected group on ED 14.5, transverse sections through the uterus horn revealed a lumen-diameter of 6 mm in average, showing the same pattern as seen on ED 13.5 and 9.5 (Figure 1B, ED 14.5, Figure 1C). Of note, the size of the uterus ampoules as analyzed by USI, was smaller compared to the mock control group with 8mm in size (Figure 1C). When we performed USI through the ampoules an undefined bright echogenic structure was visualized, indicating resorbed fetal debris (“FR”). Further detailed screening through the uterine ampoules also indicated the presence of free liquid as visualized by less echogenicity. In the majority of ampoules, no vessel-like structure was detectable by USI. While we did not detect many consolidated fetal structures in the ZIKV-infected mice, we were able to visualize the placental tissue adjacent to the resorbed fetal debris or in the liquid filled sections visible as hyperechogenic area (“P”, framed by white crosses).

In the mock-infected group, after 16 days of gestation, on ED 16.5, uterus horns could be visualized as massive anatomical structures in the USI with a mean diameter of 9.5 mm (Figure 1A, ED 16.5, Figure 1C). Transversal sections analyzing the uterus ampoules revealed a further matured fetus (“F”), as identified by defined organic structures with the highly echogenic foci identified as the fetal heart (“FC”) and the fetal paws as visualized with high echogenicity (“FP”). In addition, we could detect fully formed umbilical veins (Figure 1A, ED 16.5) connected to the associated crescent shaped placentas with bright echogenicity (“P”, framed by white crosses) lining the uterus walls.

On ED 16.5 the uterus ampoules displayed a mean of 6mm in ZIKV-infected mice (Figure 1C). In addition, we detected comparable echogenic structures indicating resorbed fetal residues as seen in prior USI (“FR”) including the placentas with brighter echogenicity (“P”, framed by white crosses) and inner side of the uterus next to a uterus lumen with less echogenic foci (“L”, in black) implying a liquid filling (Figure 1B, ED 16.5).

To exclude that mortality of the fetuses was due to the multiple handling of pregnant dams, we comparatively summarized these USI surveyed data establishing time kinetics after ZIKV challenge infection. In consequence, we observed significantly higher rates of fetal mortality in the ZIKV-infected group compared to mock-infected mice (Figure 1D). Moreover, the ZIKV-infected dams showed early signs of dead or resorbed fetuses on ED 10.5 accounting for 20% of the fetuses monitored. Subsequently, numbers of spontaneous abortion or intrauterine deaths increased over the following days resulting in almost 100% mortality on ED 16.5. However, in the PBS mock-infected group, first signs of fetal mortality were detected on ED 11.5, which constituted to 5% in the fetuses analyzed. Until ED 16.5 no increase in fetal mortality was observed confirming the ZIKV induced mortality in the fetuses.

### 3.2. Evaluation of Uteroplacental Blood Flow Activity by Doppler Ultrasound

#### Direct Comparison between ED 13.5 and ED 16.5

**Figure 2:**
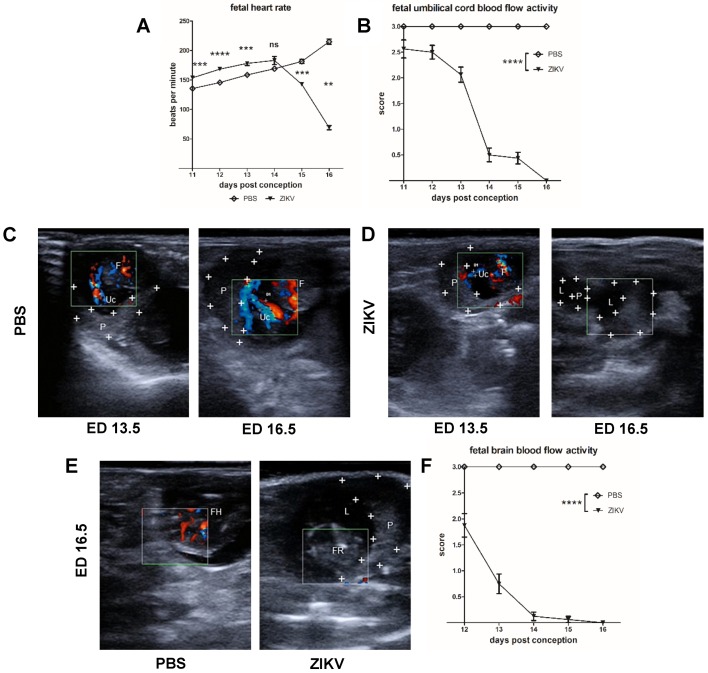
Ultrasound based demonstration of fetal blood flow activity and heart rate measured by Doppler ultrasound; (**A**) + (**B**) Pregnant interferon receptor-deficient (IFNAR-/-) mice were inoculated with PBS (mock-infected animals, used as controls) or with 10^3^ plaque-forming-units (PFU) ZIKV on embryonic day 7.5 (ED 7.5). In all experiments, heart rates of individual fetuses were monitored daily (*n* = 6-8– fetuses per group). Data are representative of two experiments. Here: Graphical summary of the data obtained by Doppler measurements of uterine vessels blood flow activities (Figure 1A: error bars indicate SEMs and differences between the groups were analyzed by unpaired, two tailed t-test. Asterisks represent statistically significant differences between two groups; *n* = ns, ** *p* = 0.0012, *** *p* = 0.0002, **** *p* < 0.0001, Figure 1B: error bars indicate SEMs and differences between the groups were analyzed by two-tailed t-test after calculation of the area under the curve, **** *p* < 0.0001); (**C**) + (**D**) Measurement of the utero-placental blood flow activity in the uterine vessels obtained by Doppler-ultrasound on ED 13.5 and 16.5 for the PBS control mice and the ZIKV-infected mice; (**E**) Measurement of the blood flow activity in the fetal heads obtained by Doppler-ultrasound on ED 16.5 for the PBS control mice and the ZIKV-infected mice; (**F**) Graphical summary of the data obtained by Doppler measurements of blood flow activities in the fetal heads (*n* = 6–8 fetuses per group); error bars indicate SEMs and differences between the groups were analyzed by two-tailed t-test after calculation of the area under the curve, **** *p* < 0.0001.

Since the data so far confirmed intrauterine growth retardation (IUGR) and fetal death in ZIKV-infected pregnant IFNAR-/- mice, we aimed to evaluate the ZIKV influence on the physiological parameters of the fetuses. For this we infected IFNAR-/- mice on ED 7.5 with ZIKV or with PBS as control and comparatively monitored the fetal heart rate in the ZIKV-infected and PBS control group starting on ED 11.5 (Figure 2A). In the PBS mock-infected group, fetal heart rate started with a mean of 145 bpm on ED 11.5 and did not significantly change until ED 14.5, showing a slight upward trend to 150 bpm (referring to mean fetal heart rate). Between ED 11.5 to ED 16.5 fetal heart rate increased to a mean of 200 bpm, corresponding to the expected physiological value [41]. ZIKV infection did not result in significantly different heart rate values (mean = 150 bpm on ED 11.5). However, it seemed that fetuses from the ZIKV-infected group showed a more distinctive increase of the heart rate between ED 11.5 and ED 14.5 indicating a mild type of fetal tachycardia. Interestingly, on ED 15.5 the fetal heart rate significantly decreased in the ZIKV-infected mice resulting in 140 bpm and on ED 16,5 fetuses only showed a rate of 50 bpm. From these data we concluded that ZIKV infection inadvertently influences the fetal circulatory system. However, since perceptible deviations on fetal heart rates were not identified before ED 15.5, we aimed also to comparatively monitor the blood flow activity in the uteroplacental vessels, which are essential for transfer of nutrients between mother and fetus (Figure 2B–D). To enable a standardized characterization of the blood flow activity pattern, we used the highly echogenic placenta as starting point to perform Doppler-US. Starting on ED 7.5, doppler ultrasonographic examination of uteroplacental circulation revealed evolving blood flow activity in the umbilical vessels. Of note, there was no obvious difference between the ZIKV-infected and mock-infected control mice until ED 13.5. We detected dynamic blood flow activity in the PBS control mice when analyzed on ED 13.5 as shown by the Color Doppler USI (Figure 2C). Doppler USI signals indicated productive recirculation of maternal blood to the fetus. Measurement of uterine artery diameter showed a mean of 0.85 mm. This is further confirmed on ED 16.5 when we detected umbilical and fetal blood vessels in comparable position. Color Doppler USI indicated that the vessel diameter was further dilated with a mean diameter of 0.93 mm. Moreover, the intensity of the signals was in line with a physiological proceeding of the pregnancy. When we performed Doppler USI in mice from the ZIKV-infected group, we were again able to specifically detect productive blood flow activity in the umbilical vessels adjacent to the placenta on ED 13.5 (Figure 2D). However, when measuring diameter of the uterine artery, we determined a mean of 0.36 mm. This diameter was smaller than the diameter in the mock control group. On ED 16.5 we again found the placenta to be a highly echogenic structure. However, on ED 16.5 we failed to detect any blood flow activity as seen by the lack of blue and red colored signals in Doppler USI. In detail, we did not observe any blood recirculation from the mother to the fetus. Moreover, confirming previous results, USI revealed undefined structures with high echogenicity indicating resorbed fetal tissue. When we summarized the data from Doppler USI, clear differences in blood flow activity were detected in ZIKV-infected dams compared to mock-infected control mice (Figure 2B). Interestingly, as early as ED 12.5 results from the Doppler USI allowed for the identification of ZIKV induced pathogenesis.

Since ZIKV infection has been associated with cerebral anomalies including microcephaly, we also assessed blood flow activity in the region of fetal head on ED 16.5 when USI allowed for differentiation of fetal structures (Figure 2E,F). In PBS mock-infected animals, color Doppler USI identified a productive blood circulation adjacent to the hyperechogenic fetal head (Figure 2E). Again, when we analyzed the Doppler USI data on the vessel diameter over time, we detected significant differences in the perfusion of fetal heads in the PBS and ZIKV-infected groups on ED 16.5 (Figure 2E). Fetuses from PBS control dams revealed an effective blood flow activity that remained stable until end of the experiment (Figure 2F). However, in the ZIKV group, fetuses showed lower perfusion rates in the heads as seen by reduced vessel diameters (44% smaller compared to PBS) on ED 12.5. Over the following days the blood flow activity in the fetal heads significantly decreased to 25% compared to the level observed in the control group on ED 13.5 and no blood flow activity was detected on ED 14.5. These data indicated that ZIKV induced pathology targeting the fetal head is much earlier detectable than in the uteroplacental transfusion.

### 3.3. Characterization of ZIKV Infection of the Placenta

**Figure 3:**
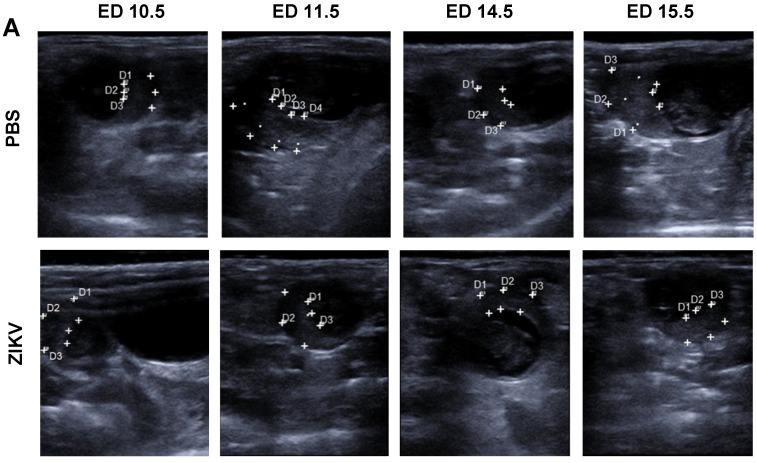

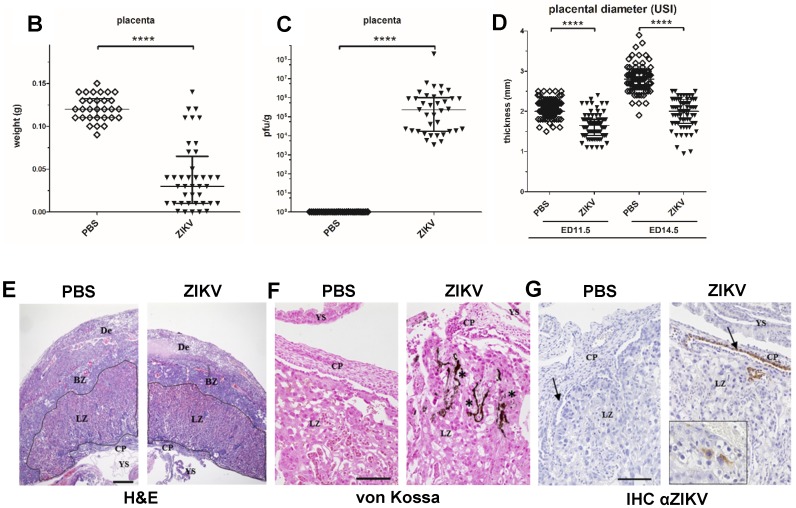
ZIKV induced changes in the placenta; (**A**) Measurement of placenta thickness by ultrasound imaging. Representative images from ED 10.5, 11.5, 14.5, and 15.5 are depicted for PBS control mice and ZIKV-infected mice; (**B**) Comparative evaluation of placental weights on ED 16.5 in sacrificed mice (*n* = 8–10 mice per group; differences between individual groups were analyzed by Mann–Whitney test. Error bars indicate the interquartile range (IQR) from the median. Asterisks represent statistically significant differences between two groups: **** *p* < 0.0001; (**C**) ZIKV challenge of pregnant mice results in highly productive ZIKV infection of placental tissues, virus titers in placentas after ZIKV infection. At the end of the experiments (ED 16.5) placentas were removed and homogenized, and the amount of virus was determined by plaque assays (*n* = 31–39 placentas per group); data are representative of at least two independent experiments; differences between individual groups were analyzed by Mann–Whitney test. Error bars indicate the interquartile range (IQR) from the median. Asterisks represent statistically significant differences between two groups: **** *p* < 0.0001; (**D**) placental diameter determined by ultrasound-based measurements comparing ED 11.5 and ED 14.5 (*n* = 31–39 placentas per group); data are representative of at least two independent experiments; differences between individual groups were analyzed by Mann–Whitney test. Error bars indicate the interquartile range (IQR) from the median. Asterisks represent statistically significant differences between two groups: **** *p* < 0.0001; (**E**–**G**) (De) decidua, (BZ) basal zone, (LZ) labyrinth zone, (CP) chorionic plate, and (YS) yolk sac. Histological analysis of placental tissues following ZIKV infection; (**E**) Placentas were removed, and sections of the organs were routinely stained with hematoxylin and eosin (HE) Bar = 300 µm; (**F**) Sections of tissues were treated with von Kossa staining to identify tissue calcification Bar = 100 µm; black asterisks indicate accumulations of dystrophic mineralization in the basal LZ of ZIKV-infected mice; (**G**) Placenta, ED 16.5, immunohistochemical staining with αZIKV. Variable, prominent intracytoplasmic antigen expression in the chorionic epithelium (black arrow), in spongiotrophoblasts and glycotrophoblasts (inset) of the ZIKV-infected group, whereas all placentas of the control group including their chorionic epithelium (black arrow) were immunonegative. Bar = 100 µm.

The placenta is considered as an important target organ for ZIKV infection resulting in direct transmission to the fetus [14,15]. Our USI data from pregnant mice demonstrated that the placenta can be detected as a clearly visible structure. To characterize the influence of ZIKV infection on the placental functionality in more detail, we comparatively monitored shape, thickness, and texture of the placenta in USI (Figure 3A). On ED 11.5, we observed no big differences in the placental thickness between ZIKV-infected and control mice with a mean thickness of 1.8 mm and 2.0 mm, respectively. Direct comparison in USI also indicated no obvious differences in the organ texture and density. However, on ED 14.5, placental thickness in ZIKV-infected mice was significantly lower than in the mock-infected control mice (Figure 3A,B). The mean thicknesses for the two groups were 1.96 mm and 2.82 mm respectively (Figure 3B). In addition, USI revealed a modified appearance of the placental texture. Placentas from ZIKV-infected mice were brighter compared to the placentas of PBS controls indicating a higher echogenicity and echotexture. In contrast, the PBS infected placentas remained visible as a crescent shape with light colored staining (Figure 3A). This data was confirmed by uteroplacental pathology including histopathological analysis at the end of the experiment. Interestingly, the absolute placental weight was significantly lower in ZIKV-infected mice compared to controls (Figure 3C). The mean placental weight in the PBS control group was 0.125 g whereas placentas from ZIKV-infected mice had a mean weight of 0.05 g only. Moreover, the histopathological examination also revealed features of ZIKV infection in agreement with the differences in thickness and echogenicity determined by USI on ED 11.5.

Finally, when monitoring virus loads, we found large amounts of virus in the placentas of ZIKV-infected mice corroborating their importance as target organ during in vivo infection (Figure 3D). Placentas of ZIKV-infected mice showed extensive ZIKV-specific staining, primarily in areas of the labyrinth zones severely affected by inflammation (Figure 3E). Several trophoblasts lines, like glycogen trophoblasts, spongiotrophoblasts, and to a lesser extent cytotrophoblasts and syncytiotrophoblasts showed antigen expression in a variable amount. Upon H&E staining, we detected large areas of densely packed inflammatory cells, mainly comprising lymphocytes and scattered macrophage-like cells as well as granulocytes in placentas from ZIKV-infected dams. Especially in the labyrinth zone, there seemed to be irregular shaped fetal capillaries in decreased numbers. Interestingly, these inflammatory processes seemed to be mainly associated in the labyrinth layer resulting in an obvious thinning of the placenta compared to mock-infected control mice (Figure 3F). Since IHC confirmed a direct ZIKV infection of placental cells, we aimed to correlate the reduced echogenicity in USI with direct effects of ZIKV infection. Of note, we specifically detected the presence of placental calcifications by Kossa-specific staining in the tissues from ZIKV-infected mice (Figure 3G). Interestingly these calcifications were mainly seen in the labyrinth zones around smaller vessels and some were filled with erythrocytes. Calcifications were visible in the basal labyrinth zone near the chorionic epithelium of infected mice, whereas in the control group this was not visible.

### 3.4. Macroscopic Outcome and Viral Burden

**Figure 4:**
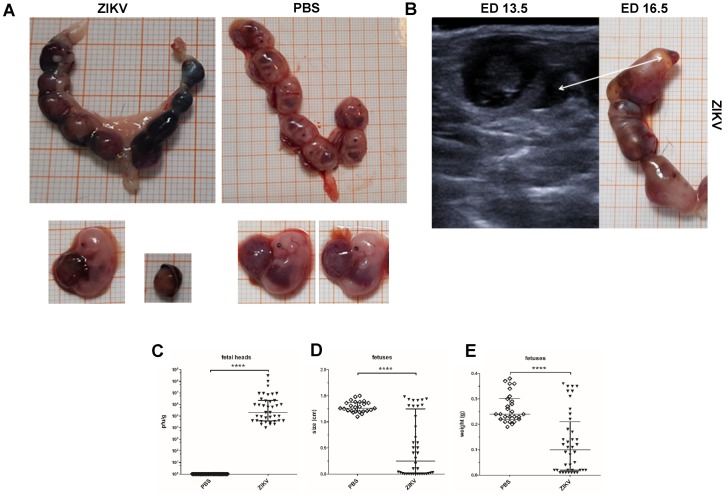

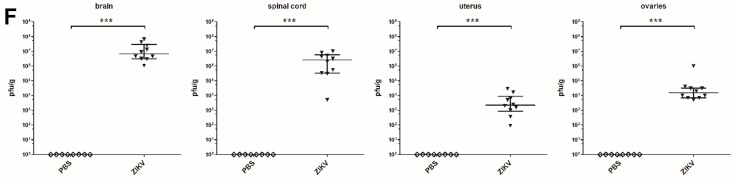
ZIKV induced pathology in the dams and the fetuses; (**A**) Comparative macroscopical evaluation of the uterus and individual fetuses from mock-infected control animals and ZIKV-infected animals at the end of the experiment on ED 16.5; (**B**) Representative ultrasound image of a ZIKV-infected uterus on ED 13.5 and the specific uterus of this ZIKV-infected animal on ED 16.5 at the end of the experiment; white arrow indicates fluid-filled areas which were already detected in USI at ED 13.5 and confirmed after necropsy; (**C**) Virus titers in the fetuses (fetal head) after ZIKV infection. At the end of the experiments (ED 16.5) fetal heads were removed and homogenized, and the amount of virus was determined by plaque assays (*n* = 31–39 per group); data are representative of at least two independent experiments; (**D**) + (**E**) ZIKV induced changes in size and weight of the fetuses as evaluated at the end of the experiment; data are representative of at least two independent experiments; (**F**) Virus titers in maternal organs (brain, spinal cord, uterus, ovaries) after ZIKV infection. At the end of the experiments (ED 16.5) organs were removed and homogenized, and the amount of virus was determined by plaque assays (*n* = 8–10 per group). Figure 4C–F: differences between individual groups were analyzed by Mann–Whitney test. Error bars indicate the interquartile range (IQR) from the median. Asterisks represent statistically significant differences between two groups: *** *p* = 0.0001, **** *p* < 0.0001; and data are representative of at least two independent experiments.

To more closely compare the results from USI to a standard characterization of ZIKV induced pathogenesis in fetuses, we euthanized the mice on ED 16.5 and performed extensive necropsy. In addition to the placental pathology we focused on the mortality, viral burden, and size of fetuses. The data so far indicated that ZIKV infection invariably leads to fetal death or resorbed fetuses until ED 14.5–15.5. In line with these data, uteri obtained from ZIKV-infected dams did not contain any viable fetuses (Figure 4A). As already visualized in USI between ED 13.5 and ED 16.5, ZIKV uteri contained multiple foci of fetal residues with the respective placenta surrounding a mass of former fetal tissue. In contrast, the uteri from mock PBS infected mothers contained multiple physiological fetuses each including the specific placenta. Of note, the ZIKV induced mortality and pathogenesis came along with massive inflammation resulting in resorption. This was seen by the presence of free liquids indicative for inflammatory processes as early as ED 13.5 (Figure 4B). Interestingly, we confirmed inflammation in necropsy on ED 16.5. When we characterized the structure of fetuses in more detail, mean length of fetuses was significantly smaller in the ZIKV-infected group compared to the control group (Figure 4D). Here, PBS control fetuses showed a mean size of 1,25 cm whereas the fetuses from ZIKV-infected animals had a mean of 0.5 cm. Moreover, the fetuses from the ZIKV-infected dams also showed significantly lower total weights with a mean of 0.11 g compared to 0.29 g in the viable fetuses from the PBS control group (Figure 4E). To determine whether fetal damage goes along with direct ZIKV infection in the fetus, we analyzed ZIKV infectious virus in fetal heads by plaques titration (Figure 4C). We found large amounts of virus with a mean of 10^7^ PFU/g tissue in the ZIKV group. As expected, we failed to detect ZIKV in the PBS control group. To confirm productive infection in the dams, we also detected ZIKV in the brain and spinal cord, the main target organs of the virus (Figure 4F). Here, ZIKV-infected mice had high viral titers in the brain (10^7^ PFU/g tissue) and the spinal cord with a mean of 5 × 10^6^ PFU/g tissue. In addition, we also evaluated the viral loads in the ovaries and uteri to estimate the general impact of the infection on the reproductive tract (Figure 4F). Interestingly, we detected lower amounts of ZIKV in these organs compared to brain and spinal cord, with a mean of 10^4^ PFU/g tissue for the uterus and a mean of 8 × 10^5^ PFU/g tissue in the ovaries indicating a placenta derived transmission of blood borne ZIKV to the fetuses.

## 4. Discussion

ZIKV infection during pregnancy has been confirmed to lead to severe congenital malformations and intrauterine death in human fetuses. During the 2015/2016 ZIKV epidemic, microcephaly was the predominant symptom of congenital ZIKV infection in newborns. So far, there are no vaccines or licensed therapeutics available. As a result, preclinical studies in mouse models of ZIKV infection during pregnancy are essential to evaluate therapeutic and preventive measurements. A major hurdle of such studies is to distinguish between ZIKV pathogenesis and immunopathology. Thus, we aimed to establish a new diagnostic approach to directly and rapidly follow the infection in fetal IFNAR-/- mice. In this study we established USI as imaging tool to evaluate ZIKV induced pathology in fetal mice in vivo during pregnancy. Using USI in pregnant IFNAR-/- mice, we demonstrated fetal growth retardation and fetal death over time, indicating maternal-fetal transmission of ZIKV after infection on ED 7.5. In pregnant women, obstetric sonography is well established as safe diagnostic tool with clinical utility, which is supported by numerous human case reports and epidemiological studies. For ZIKV suspected cases, CDC recommends repeated diagnostic ultrasound imaging (every 3–4 weeks) to evaluate fetal anatomy, particularly the head circumferences and the growth (https://www.cdc.gov/pregnancy/zika/testing-follow-up/prenatal-care.html). However, obstetric sonography is not commonly used to examine pregnant mice due to their short gestation time and the smaller size. For some other experimental approaches e.g., brain development studies or evaluation on mouse social behavior, USI on pregnant mice has been already used as valuable tool [42,43]. The overall goal of our study was to contribute to the development of USI as for the monitoring of congenital infections in mice. We chose ZIKV as a model pathogen, and to best of our knowledge USI has not been tested as rapid diagnostic tool in a mouse model of ZIKV infection. Indeed, there are already published studies characterizing murine models of congenital Zika virus syndrome with regard to placental and fetal pathologies. Our objective was to analyze the course and outcome of ZIKV infection in the IFNAR-/- mouse model. Different mouse models have been established as preclinical models to better understand ZIKV induced pathogenesis. Thus, mouse models also offer the opportunity to study influence of ZIKV infection for the fetuses using pregnant mice. Of course, mice are different from humans with regard to physiology and disease development. In detail, mice reveal a significantly shorter gestation time and the placental layers are different compared to humans. However, mice and humans both feature a hemochorial placenta [44,45]. From that aspect mouse studies might be appropriate to evaluate pathogenesis and countermeasures of congenital pathogens. As established by Miner et al. [30], ZIKV infection in pregnant IFNAR-/- mice results in a slightly different clinical outcome compared to the human ZIKV pathogenesis, since the fetuses do not show microcephaly, brain calcification and defects in brain development. However, this feature does not hamper the definition of fetoprotective countermeasures in the IFNAR-/- pregnancy model. Miner et al. found a profound pathological change in ZIKV-infected placentas. Here, the hypothesis is that ZIKV infects the fetus through blood-placental transmission causing direct viral cytotoxicity. In addition, the fetus is also damaged due to the destruction of the placenta resulting in severe ischemia [46]. For our approach to study the effects of candidate vaccines or therapeutics to prevent ZIKV transmission to the fetus, this model might be well-suited. In the context of USI characterization, the first step was to visualize ZIKV induced changes in the fetus and in the placenta. Our results from daily USI of pregnant ZIKV-infected and mock-infected pregnant mice demonstrated that first visible signs of infection were observed on ED 13.5, which corresponds to the second trimester in human pregnancy. This is in line with the results from case reports in humans. Here, after potential ZIKV infections during pregnancy, ultra-sonographic examination also identified first signs of ZIKV pathogenesis in the second trimester [17]. The leading symptom of ZIKV pathogenesis as evaluated in obstetric sonography was the growth retardation. This was also confirmed in our USI-mouse model. The sonography allows to specifically visualize fetal development and growth, as we were able to differentiate between the respective fetuses and the corresponding body parts. For this, fetal USI in mice might also contribute to other aspects of research questions involving pregnant mice. Of note, we did not detect distinct fetal structural abnormalities in single body parts. Instead we observed significant fetal growth retardation compared to the mock-infected fetuses. This obvious clinical parameter might allow to rapidly confirm fetoprotective countermeasure also in other applications. In this first approach, we did not observe significant modifications in the gross-morphological structure of the placentas from ZIKV-infected dams. This is different from the human case reports, where placentas already revealed visible calcification in the second trimester even though the placental thickness appeared normal [17]. Placenta pathology, e.g., calcifications, is likely more obvious in humans due to the comparatively longer gestation time. Thus, the extent of the calcifications is more advanced and better visible by USI. In addition, most of the human pregnancy comprises a single pregnancy. In contrast, mice harbor multiple pregnancies including multiple placentas. Thus, the USI analysis is more difficult in mice. Nevertheless, using histological methodology we could readily confirm mineralization of placental tissue in mice. Thus, the ZIKV mouse model is still to be considered as an appropriate model to study these pathological processes also due to the similar type of placentas (humans and mice both harbor a hemochorial placenta). However, when we specifically monitored the placental anatomy in more detail, the placenta thickness appeared to continuously decrease over the time starting about ED 11.5. This shrinking indicates a ZIKV induced pathological conversion of the physiological placental tissue in favor of calcifications. This has been already described in previous studies [47]. Here, placental thinning as identified by echogenic foci correlated with calcification of the placenta. This is further confirmed by the post-mortem analysis of the placentas confirming that the significantly lower weights are a result of continued calcification. Placental calcification is an also common phenomenon in physiological pregnancy at term and is regarded as a physiological aging process [48,49]. However, earlier placental calcification could be considered as a direct cytotoxic effect of ZIKV infection. Indeed, we detected significant amounts of ZIKV in placentas. This is in line with data from Hastings and colleagues who analyzed the influence of TAM receptors for the transmission of ZIKV through the transplacental route and replication of the virus in fetal tissues in the pregnant ZIKV mouse model [50]. Moreover, productive placental ZIKV infection, as visualized in USI, was further confirmed in gross morphological analysis. This is in line with the data from other preclinical ZIKV models. In pregnant rhesus maquaces, Hirsch et al. [37] showed placental infarctions with large gross vascular obliteration and villous stromal calcifications, which were a consequence of stromal fibroblast cell death. Another outcome of our placental ZIKV infection model was a reduced uteroplacental blood flow activity, which was measured using Doppler-USI. Interestingly, this observation appeared to be the most obvious and earliest sign of ZIKV pathogenesis when evaluating the course of disease by USI. Of note, the contribution of vascular restriction on fetal and placental growth on IUGR and decreasing cardiac output has already been confirmed in human studies [51,52]. Since these alterations were already visible as early as ED 12.5 in the fetuses, this will allow for the evaluation of fetoprotection with a high sensitivity. In addition, using Doppler USI monitoring, as established in the ZIKV mouse model, the identification of possible effects of immunopathogenesis in control mice will be possible. To date we have a very limited understanding about the kinetics of immune responses during pregnancy. For example, we have limited knowledge of the mechanisms of vaccine-induced protection for the fetus after vaccination of the mother. This is also true for microbial defense during pregnancy. In this context, the placenta is known to be an active immunological organ orchestrating innate immune responses to protect the fetus by secretion of inflammatory cytokines. The type of reactivity initiated by the placenta dictates the outcome of the immunological response for the fetus and the mother [53]. Thus, the availability of an imaging tool that allows for the classification of substances that are harmful and not harmful to fetuses has the potential to be valuable for other applications. We used this imaging tool to monitor blood flow activity in the fetal brain. Using this approach, we were able to detect visible differences between the mock-infected and ZIKV-infected group. However, a real correlation was not possible, as the profound effect of ZIKV infection on fetuses made it impossible to differentiate and locate the fetal heads by USI. This is in line with previous studies from Miner et al. In the mouse model, they did not detect ZIKV induced microcephaly as the major clinical outcome in newborn. This might be due to neurodevelopmental differences during embryogenesis in mice and humans [30]. In addition, our data indicate that ZIKV infection results in significant IUGR which in consequence probably induces efficient fetal resorption. This hypothesis would also explain the absence of microcephaly in the mouse model. In contrast, in the non-human primate model, ZIKV infection during pregnancy results in microcephaly and brain defects [54]. Of note, the hypothesis of rapid fetal remission is further supported by the findings of post-mortem evaluations. This has been confirmed in the uterus, where we revealed free liquids in USI indicative for massive reactivity and reorganization due to ZIKV induced pathogenesis. In summary, our USI based characterization of ZIKV disease in pregnant IFNAR-/- revealed the suitability of this method to identify and correlate infection with pathogenesis. This is because we could directly confirm the findings as evaluated during the course of infection by USI with the results obtained after necropsy, which included viral titration and macroscopical findings. In future studies it will be interesting to combine such models of congenital ZIKV infection with USI and/or therapeutic countermeasures. Miner and Coyne used the pregnant mouse model to comparatively evaluate congenital infection and placental and fetal pathology [55]. In the next step they transferred the results from the mouse model to human placental explants. This approach will be also appealing to study other infections in the pregnant mouse model using USI/ US Doppler as intra vitam readout, e.g., other flaviviruses, murine cytomegalovirus, or toxoplasma gondii [56,57]. Future work in this model will be particularly helpful to further analyze the influence of selected therapeutic and preventive agents on the outcome of infection in fetuses and mothers.

## Figures and Tables

**Table:** (**a**)

**Pulsating Umbilical Cord Diameter**						
**Score “a”**	ED 11.5	ED 12.5	ED 13.5	ED 14.5	ED 15.5	ED 16.5
3	>0.5 mm	>0.5 mm	>0.6 mm	>0.6 mm	>0.7 mm	>0.8 mm
2	<0.5 mm	<0.5 mm	<0.6 mm	<0.6 mm	<0.7 mm	<0.8 mm
1	<0.3 mm	<0.3 mm	<0.4 mm	<0.4 mm	<0.5 mm	<0.5 mm
0	Neg.	Neg.	Neg.	Neg.	Neg.	Neg.

**Table:** (**b**)

**brain vessel diameter**					
**Score “b”**	ED 12.5	ED 13.5	ED 14.5	ED 15.5	ED 16.5
3	>0.05 mm	>0.05 mm	>0.05 mm	>0.08 mm	>0.08 mm
2	<0.05 mm	<0.05 mm	<0.05 mm	<0.08 mm	<0.08 mm
1	<0.03 mm	<0.03 mm	<0.03 mm	<0.05 mm	<0.05 mm
0	Neg.	Neg.	Neg.	Neg.	Neg.

**Table:** (**c**)

**Score “c”**	**% living fetuses**
3	>80%
2	<80%
1	<50%
0	<25%

**Scoring**: umbilical cord blood flow activity = (Score “**a**” + Score “**c**”) / 2; fetal brain blood flow activity = (Score “**b**” + Score “**c**”)/2.
